# Molecular detection of HPV, EBV, and polyomaviruses in thyroid tumors and their clinicopathological relevance

**DOI:** 10.1007/s00432-025-06328-1

**Published:** 2025-10-20

**Authors:** Nagwan Ramadan, Omar Alfarouk Rabiee, Mohamed M. Hafez, Nasra F. Abdel Fattah, Eman Naguib Khorshed, Khaled Khalafalla, Auhood Nassar

**Affiliations:** 1 Department of Microbiology, Faculty of Science, Ain Shams University, Cairo, Egypt; 2 Virology and Immunology Unit, Cancer Biology Department, National Cancer Institute, Cairo University, Cairo, Egypt; 3 Department of Surgical Pathology, National Cancer Institute, Cairo University, Cairo, Egypt; 4 Department of Nuclear Medicine, National Cancer Institute, Cairo University, Cairo, Egypt

**Keywords:** Human Papillomavirus, Epstein Barr virus, Human polyomavirus, JCV, BKV, Thyroid Cancer

## Abstract

**Objective:**

Oncogenic viruses have been implicated in thyroid carcinogenesis, yet their prevalence and clinicopathological associations remain incompletely understood. Thus, it is crucial to investigate the prevalence of human papillomavirus (HPV), Epstein–Barr virus (EBV), and polyomaviruses (JCV and BKV) in thyroid tumors and assess their association with clinic-pathological characteristics.

**Methods:**

This study included 70 fresh biopsy samples collected from 45 TC patients and 25 patients with benign thyroid tumors, along with 10 normal thyroid tissues. Viral DNA was extracted and screened for HPV, EBV, and polyomaviruses using SYBR Green–based real-time PCR.

**Results:**

HPV, EBV, and polyomaviruses, particularly JCV, were detected at significantly lower frequencies in the normal group when compared to malignant and benign groups (*p*-value = 0.030, *p*-value = 0.030, and *p*-value = 0.001, respectively). In TC patients, HPV common, HPV-16, HPV-6, and HPV-11 positivity was correlated with obesity (*p*-value < 0.05), polyomaviruses, particularly JCV, with older age (*p*-value = 0.041 and *p*-value = 0.011), BKV with larger tumor size (*p*-value = 0.030), and EBV with family cancer history (*p*-value = 0.020). In benign tumors, polyomavirus was absent in Hashimoto thyroiditis (*p*-value = 0.020), BKV was linked to older age (*p*-value = 0.030), and absence of BKV was associated with COVID-19 vaccination (*p*-value = 0.046).

**Conclusion:**

The current study is the first of its kind in Egypt to investigate the prevalence of HPV, EBV, and polyomaviruses in thyroid tumors and to examine their associations with certain clinicopathological characteristics. The findings underline the importance of viral profiling in understanding thyroid tumor behavior and influencing cancer risk as well.

**Supplementary Information:**

The online version contains supplementary material available at 10.1007/s00432-025-06328-1.

## Introduction

Thyroid cancer (TC) is recognized as the most prevalent cancer of the endocrine system. It typically originates from follicular epithelial cells (Bhaijee and Nikiforov 2011). It can be classified as well-differentiated Papillary Thyroid Carcinoma (PTC) which is the most common type of TC, accounting for approximately 80% of cases (Nikiforov 2011). PTC is characterized by its papillary structures and nuclear features, such as overlapping, clearing, and grooves (Kitahara and Sosa 2016; Reuters et al. 2018). Follicular Thyroid Carcinoma (FTC) is the second most common subtype which represents about 15% of TCs and arises from follicular cells (Nikiforov 2011). Globally, the incidence of TC has significantly increased, making it ranks the ninth of all cancers. In 2020, about 3.2 million people had TC with approximately 586,202 new cases. TC usually occurs at the age of 32–65 years and is significantly more incident in women than in men. It ranks as the fifth of all cancers in women and the sixteenth in men. In Egypt, TC ranks as the thirteenth by 2% of all cancers (Worldwide Cancer Data 2024; IARC 2025; Pizzato et al. 2022).

In recent years, increasing attention has been directed toward the possible role of viruses in the development of various human cancers (Javier and Butel 2008). Most human cancer viruses act as initiators or promoters in the oncogenic process (Järviluoma and Ojala 2006). Each virus tends to have specific cellular targets, despite sharing similar general pathways involved in disrupting cellular regulation (Greene et al. 2007). The correlation between viral infection and PTC has been confirmed by several studies, based on the presence of viral genome in the tumor tissues (Bhaijee and Nikiforov 2011). Human Papillomavirus (HPV), a non-enveloped DNA tumor virus, is primarily linked to cervical and head and neck cancers (Guo et al. 2018; Wallin et al. 1999; Yang et al. 2019). High-risk types, particularly HPV 16 and 18, account for 3% of malignancies in women and 2% in men (Economopoulou et al. 2020; Senkomago et al. 2019). HPV 6 and 11, the low-risk subtypes, usually linked to benign papillomas and have also been found in thyroid tissues (El Moussaoui et al. 2021; Trzcinska et al. 2020). The prevalence of HPV in the PTC tissues was reported to be significantly more than the benign thyroid nodules (Archin Dialameh et al. 2021). Another study investigated the presence of HPV DNA in nodular thyroid diseases and revealed its absence neither in nodular nor normal thyroid tissue (Stamatiou et al. 2015). Although many studies revealed the detection of HPV DNA in thyroid tumors (Archin Dialameh et al. 2021; Mostafaei et al. 2020a) no definitive causative role of HPV in TC has been established.

Epstein-Barr virus (EBV), a herpesvirus causing infectious mononucleosis, is strongly linked to many cancers (Thorley-Lawson and Gross 2004; White and Fenner 1994). EBV DNA and its EBNA1 gene have been identified in TC tissues. One study found EBNA1 in PTC patients, with higher prevalence in younger individuals when compared to EBV-negative patients (Homayouni et al. 2017). Some investigations have confirmed the link between the EBV and the incidence or development of TC, demonstrating not only the role of its genes in causing the tumor but also the morphological and molecular changes which induced in the thyroid neoplastic cells (Almeida et al. 2019, 2020; Bychkov and Keelawat 2017; Golden et al. 2017; Homayouni et al. 2017; Moghoofei et al. 2019).

Few studies have investigated the relationship between polyomaviruses (PyVs) and TC (Karimi et al. 2022; Stamatiou et al. 2015, 2016; Vivaldi et al. 2003). JCV is a widespread polyomavirus infecting most people in childhood (Tan et al. 2019). While often asymptomatic, it has been associated with multiple cancers (Enam et al. 2002; Sinagra et al. 2017). In a TC cohort, the presence of JCV DNA in a subset of PTC samples was confirmed, suggesting a potential association between JCV and thyroid carcinogenesis (Karimi et al. 2022). BKV is another polyomavirus known for causing nephropathy in kidney transplant recipients (Hirsch and Randhawa 2013; Wang et al. 2022). However, its early genome has also been detected in various cancers (Abend et al. 2009; Levican et al. 2018). In a study conducted on PTC Iranian patients, the presence of BKV DNA in a subset of PTC samples was identified (Tarharoudi et al. 2022). Although viral infection is not the only mediator responsible for TC development, it might be considered a contributing aspect in our study. Further research is needed on the involvement of virus infection in the development of TC. The current study is the first of its kind in Egypt to investigate the association between TC and multiple viruses, along with providing an initial look into the potential impact of COVID-19 infection and vaccination on TC incidence.

## Methods

### Patients and sample collection

The present study was conducted on 70 fresh biopsy samples collected from patients with thyroid tumor who were diagnosed and treated at the Medical and Surgical Oncology Department, National Cancer Institute (NCI), Cairo University, Egypt, from May 2024 to January 2025. Patients were divided into 2 groups; 45 patients with TC and 25 patients with benign thyroid tumor, in addition to 10 normal samples serving as the control group. Twenty out of forty-five patients received RAI-131 therapy at Nuclear Medicine Department, NCI, Cairo University, Egypt. The Institutional Review Board (IRB) of the NCI approved the study (IRB approval number: CB2420-102-056), and written informed consent was received from all participants who joined the study.

### Patients’ data

The patients’ data were retrieved from their medical records maintained by the Biostatistics and Epidemiology Department, as well as from the Nuclear Medicine Department, NCI, Cairo University. Also, the pathological data were collected under the supervision of the pathologist in the Pathology Department, NCI, Cairo University.

### Specimen collection

A total of 250 mg of fresh tissue biopsy was collected from patients with malignant and benign thyroid tumors, as well as 10 control subjects. All tissue samples were stored at − 80 °C till being used.

### Nucleic acid extraction

Viral DNA was extracted using QIAamp DNA extraction Mini kit (Qiagen, Germany: Cat. No. 51304) following the manufacturer’s instructions. the quality and quantity of isolated viral DNA were assessed using a Nano-Drop 2000 spectrophotometer (Thermo Fisher Scientific). The extracted DNA was stored at − 80 °C till further analysis.

### Detection of high- and low- risk HPV genotypes, EBV, and polyomaviruses by syber green- based real-time PCR

For contamination-free quantitative PCR analysis, certain precautions were followed. These included wearing proper personal protective equipment (PPE), working in separated areas, and ensuring all equipment and working surfaces were regularly decontaminated. Furthermore, reagents were dispensed into aliquots and kept away from samples. Positive and negative controls were validated, and results were carefully reported.

For HPV detection, the HPV Late 1 (L1) region was used as a target for PCR amplification using common primers. For specific detection of high- and low- risk HPV genotypes, genotype- specific primers were used to target HPV-6, 11, 16, 18, 33, and 58. Moreover, EBV DNA was detected by targeting the nucleotide positions 32,104 to 32,256 bp of the BamH1 W genomic region (Accession AB850660). For polyomaviruses DNA detection, the large T genomic region of Polyomavirus was targeted. In the first round of amplification, the nucleotide positions 3978 to 4150 of the JCV genome (Accession AF281613) were amplified, followed by a semi-nested PCR targeting nucleotide positions 3978 to 4048 of the JCV genome. Similarly, BKV- specific primer was used to detect BKV genome in a semi-nested PCR assay.

Positive and negative (non- template) controls were included in each assay. For HPV positive control, DNA was extracted from Michigan Cancer Foundation-7 (MCF7) cells (ATCC HTB-22), which were sourced from the Egyptian company VACSERA (Metwally et al. 2021). For EBV, we used established positive controls from the American Type Culture Collection (ATCC). The EBV control was the ATCC strain VR-1492. The positive controls for the polyomaviruses (BKV and JCV), strains VR-837 and VR-1583 respectively were also obtained from the ATCC (Metwally et al. 2021).

For all molecular assays, the amplification was conducted in a final volume of 20 μL of 1 × Perfect Start® Green qPCR Super Mix kit (TransGen Biotech, Beijin, China), 1 × universal passive reference dye, 0.2 μM of forward and reverse primer, and 100 ng of extracted DNA, using nuclease-free water to make up the volume. The primer sequences used in the molecular assays are presented in Table 1. For HPV, the cycling conditions were performed as in the previously published protocol by Metwally et al. (Metwally et al. 2021), using the quantitative real-time detection system (Applied Biosystems Step One Real-Time PCR system Thermal Cycling Block, USA). For EBV and polyomaviruses, thermal cycling was initiated with incubation at 94 °C for 30s, then 40 cycles of 94 °C for 5s, 55 °C for 15s, and 72 °C for 10s, at the end of which fluorescence was read. The threshold cycle (Ct) value for each sample was detected. Post amplification melting curve analysis (Tm) along with gel electrophoresis, clearly differentiated the specific viral product sequences from non-specific PCR products.

**Table 1:** List of Primer Sequences used for HPV, EBV, and Polyomaviruses Detection

Virus	Sequences
HPV common	(MY11): 5ʹCGT CCM ARR GGA WAC TGATC3ʹ
	(MY09): 5ʹGCM CAG GGW CAT AAY AATGG3ʹ
HPV-6/11	5ʹTGCAAGAATGCACTGAACAC3ʹ
	5ʹTGCATGTTGTCCAGCAGTGT3ʹ
HPV 16	5ʹGGCTCTGGGTCTACTGCAAA3ʹ
	5ʹTTCCTCCCCATGTCGTAGGT3ʹ
HPV-18	5ʹGCATAATCAATTATTTGTTTACTGTGGTAGA3ʹ
	5ʹCCTATACTGCTTAAATTTGGTAGCATCATA3ʹ
HPV-33	5ʹACTATACACAACATTGAA3ʹ
	5ʹGTTTTTACACGTCACAGTGCA3ʹ
HPV-58	5ʹGTAAAGTGTGCTTACGATTGC3ʹ
	5ʹGTTGTTACAGGTTACACTTGT3ʹ
EBV	5ʹCAC TTT AGA GCT CTG GAG GA3ʹ
	5ʹTAA AGA TAG CAG CAG CGC AG3ʹ
Polyomavirus F	5ʹAAG TCT TTA GGG TCT TCTAC3ʹ
Polyomavirus R	5ʹ GTG CCA ACC TAT GGA ACAGA3ʹ
JCV	5ʹ TGA TGA AAA CAC AGG ATCC3ʹ
BKV	5ʹ GAG TCC TGG TGG AGT TCC3ʹ

### Statistical methods

Data management and analysis was performed using Statistical Package for Social Sciences (SPSS) vs. 26. Numerical data were checked for normality and were statistically described as means (standard deviations) or median (range) as appropriate. Categorical data were described as numbers and percentages. Numeric data was analyzed using independent t-test for normal data and Mann–Whitney U test for non-normally distributed data, while comparison of more than two groups, ANOVA was used for normal data and Kruskal–Wallis test for non-normally distributed data. Chi-square or Fisher's exact test was used to examine qualitative variables as appropriate. Multiple Logistic regression analysis was performed for determining independent variables. All tests were 2 tailed and *P*-value < 0.05 was considered statistically significant.

## Results

### Clinico-pathological findings

PTC was the most common histological type among the studied TC cases, followed by FTC, medullary, Hurthle cell, and anaplastic carcinomas. The median tumor size was 2 cm, ranging from 0.3 to 12.0 cm. Regarding tumor grade, the classic variant was the most predominant one (60.0%). Most tumors were unifocal and unilateral (73.3 and 77.8%, respectively). Lymph node involvement was present in 48.0% of patients and T1a was the most frequent T-stage (27.5%). All clinico-pathological findings of TC patients are presented in Table 2**.**

**Table 2:** Clinico-pathological findings of thyroid cancer patients

Thyroid Cancer Patients (n = 45)		N	%
Tumor type	Papillary carcinoma	35	77.8
	Follicular carcinoma	4	8.9
	Medullary carcinoma	1	2.2
	Hurthle cell carcinoma	1	2.2
	Anaplastic carcinoma	1	2.2
	NIFTP	3	6.7
Tumor size^*^	Median (range)	2	(0.3–12.0)
Grade type	Angioinvasive	1	2.2
	Classic	27	60.0
	Follicular	7	15.6
	High grade	1	2.2
	Minimally invasive	1	2.2
	NA	5	11.1
	Well differentiated	1	2.2
	Widely invasive	2	4.4
Focality	Unifocal	33	73.3
	Multifocal	12	26.7
Laterality	Unilateral	35	77.8
	Isthmus	2	4.4
	Bilateral	8	17.8
Lymph Nodes	Negative	13	28.9
	Positive	12	26.7
	NA	20	44.4
T-stage	T1a	11	24.4
	T1b	7	15.6
	T2	8	17.8
	T3a	9	20.0
	T3b	5	11.1
	NA	5	11.1
N-stage	N0	13	28.89
	N1	12	26.67
	Nx	17	37.78
	NA	3	6.66

^*^ 1 missing case for tumor size

### SYBR green–based real-time PCR amplification and melting curve plots of HPV, EBV, and polyomaviruses

**Fig. 1:**
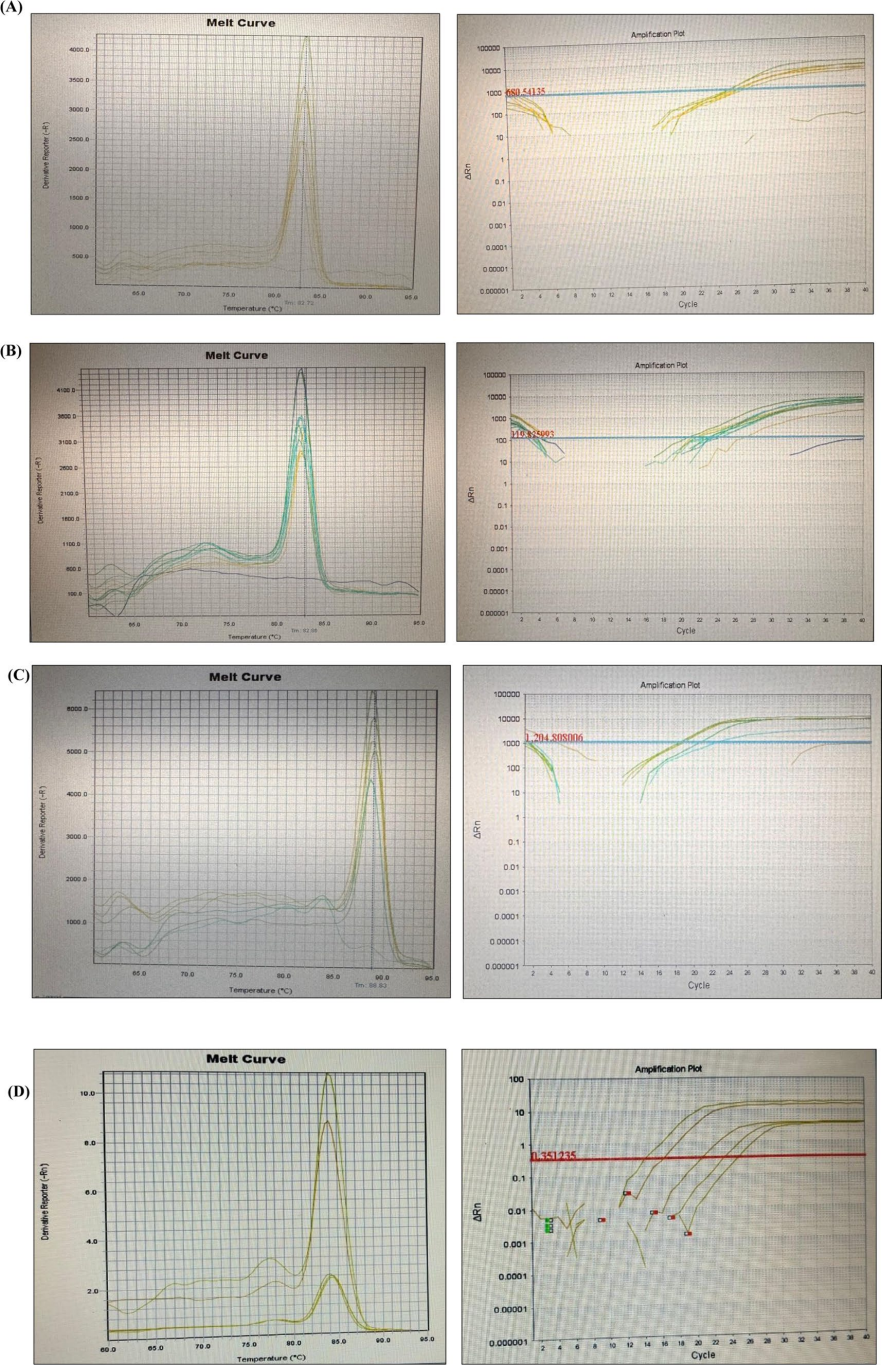

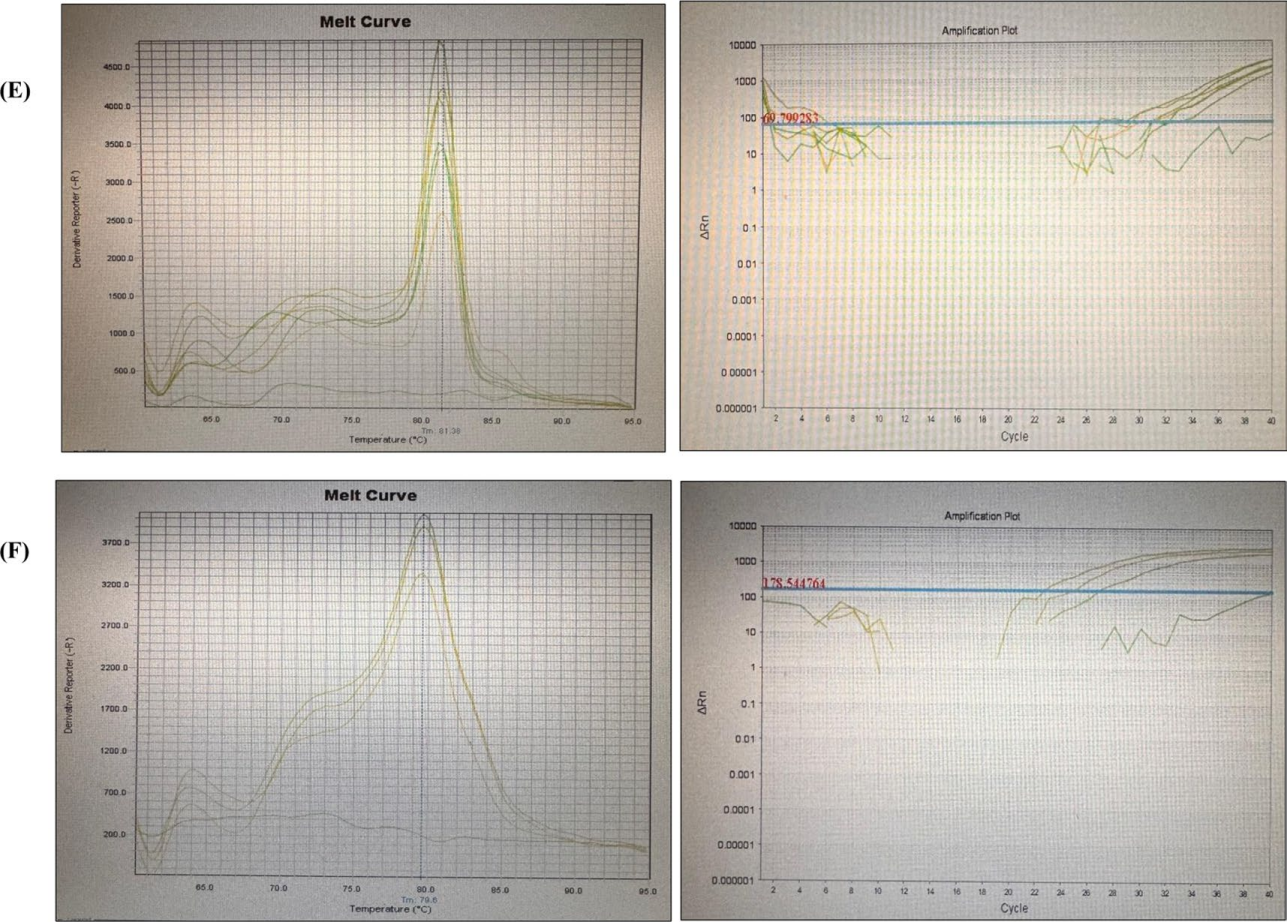
Amplification and melting curve plots of **A** HPV-18, **B** HPV-16, **C** HPV-6/11, **D** EBV, **E** JCV, and **F** BKV

**Fig. 2:**
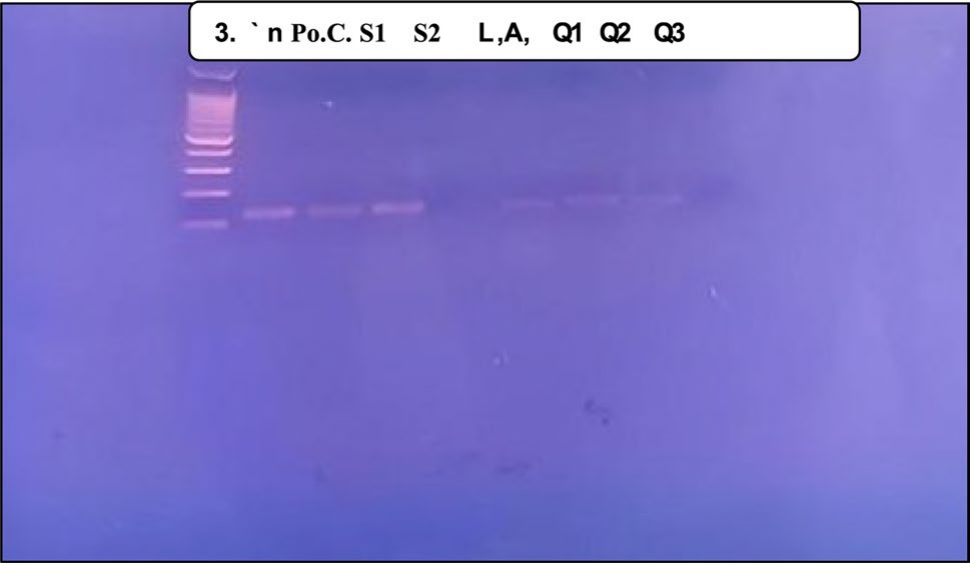
Agarose gel electrophoresis of the amplification of the target region of JCV genome. Abbreviations; Po. C.: positive control, N.C.: negative control (non-template), S: sample

Amplification and melting curve plots of HPV, EBV, and Polyomaviruses are presented in Fig. 1. No amplification was observed in negative controls (non-template), indicating absence of contamination. Melting curve analysis confirmed the specificity of the amplification products. Each positive amplification showed a single distinct peak at 82.7 °C for HPV-18, 82.8 °C for HPV-16, 88.83°C for HPV-6/11, 84.0 °C for EBV, 81.3 °C for JCV, and 79.6 °C for BKV. No additional peaks were observed, indicating absence of non-specific amplification. However, shoulder formation was detected in the melting curves of positive samples of JCV. Thus, the qPCR products were further confirmed by agarose gel electrophoresis. Clear bands matching with positive control and corresponding to the expected amplicon size were identified and presented in Fig. 2.

### Association of viral profile and clinicopathological characteristics in thyroid cancer patients

In the present study, several significant associations were observed between viral presence and clinicopathological features in TC patients. Table 3 presents the association of HPV subtypes with clinicopathological characteristics in TC patients. HPV common, HPV-16, HPV-6, and HPV-11 viruses showed statistically significant correlations with obesity (*p*-value = 0.019, *p*-value = 0.043, and *p*-value = 0.029, respectively). Additionally, positivity of Polyomaviruses and JCV were associated with older age (*p*-value = 0.041 and 0.011, respectively) (Table 4). Interestingly, BKV positivity was significantly linked to the larger tumor size (*p*-value = 0.03) with a median = 3.3 vs. 1.5 cm. Notably, presence of EBV was significantly more frequent in patients with familial cancer history (*p*-value = 0.020) as shown in Table 5. These findings highlight the significance of viral profiling in understanding tumor behavior, especially in patients with specific clinical conditions such as obesity, older age, or family history of cancer.

**Table 3:** Association of HPV subtypes with clinicopathological characteristics in thyroid cancer patients

		HPV common				*p*-value ^a^
		Negative (n = 25)		Positive (n = 20)		
		n	(%)	n	(%)	
Age, mean (SD)		44	11	46	12	0.467^c^
Gender	Male	6	54.5	5	45.5	0.938
	Female	19	55.9	15	44.1	
Tumor type	Papillary carcinoma	19	54.3	16	45.7	–
	Follicular carcinoma	3	75.0	1	25.0	
	Medullary carcinoma	1	100.0	0	0.0	
	Hurthle cell carcinoma	1	100.0	0	0.0	
	Anaplastic carcinoma	0	0.0	1	100.0	
	NIFTP	1	33.3	2	66.7	
Tumor size, median (range)		2.0	(0.3–6.5)	1.8	(0.3–12.0)	0.850^d^
Grade Type	Angioinvasive	0	0.0	1	100.0	–
	Classic	13	48.1	14	51.9	
	Follicular	5	71.4	2	28.6	
	High grade	0	0.0	1	100.0	
	Minimally invasive	1	100.0	0	0.0	
	NA	3	60.0	2	40.0	
	Well differentiated	1	100.0	0	0.0	
	Widely invasive	2	100.0	0	0.0	
T-stage	T1a	7	63.6	4	36.4	–
	T1b	4	57.1	3	42.9	
	T2	3	37.5	5	62.5	
	T3a	5	55.6	4	44.4	
	T3b	3	60.0	2	40.0	
N-stage^†^	N0	8	61.5	5	38.5	0.320
	N1	5	41.7	7	58.3	
	Nx^†^	9	52.9	8	47.1	
Focality	Unifocal	20	60.6	13	39.4	0.258
	Multifocal	5	41.7	7	58.3	
Laterality	Unilateral	22	62.9	13	37.1	–
	Isthmus	1	50.0	1	50.0	
	Bilateral	2	25.0	6	75.0	
Lymph Nodes	Negative	8	61.5	5	38.5	0.320
	Positive	5	41.7	7	58.3	
Family History	No	8	57.1	6	42.9	0.923
	Yes	15	55.6	12	44.4	
Previous cancer	No	19	59.4	13	40.6	0.601
	Yes	5	50.0	5	50.0	
Chemotherapy	No	22	57.9	16	42.1	1.000^b^
	Yes	2	50.0	2	50.0	
Radiotherapy	No	22	59.5	15	40.5	0.636
	Yes	2	40.0	3	60.0	
Obesity	Normal	9	90.0	1	10.0	0.019 ^b^ *
	Overweight	7	63.6	4	36.4	
	Obese	8	38.1	13	61.9	
Chronic thyroiditis (e.g., Hashimoto's thyroiditis)	No	22	57.9	16	42.1	0.640^b^
	Yes	2	40.0	3	60.0	
Covid 19	No	18	58.1	13	41.9	0.839
	Yes	6	54.5	5	45.5	
Covid 19 vaccine	No	9	64.3	5	35.7	0.508
	Yes	15	53.6	13	46.4	

		HPV-16				*p*-value ^a^
		Negative (n = 36)		Positive (n = 9)		
		n	(%)	n	(%)	
Age, mean (SD)		45	12	45	10	0.991^c^
Gender	Male	10	90.9	1	9.1	0.298
	Female	26	76.5	8	23.5	
Tumor type	Papillary carcinoma	27	77.1	8	22.9	–
	Follicular carcinoma	4	100.0	0	0.0	
	Medullary carcinoma	1	100.0	0	0.0	
	Hurthle cell carcinoma	1	100.0	0	0.0	
	Anaplastic carcinoma	1	100.0	0	0.0	
	NIFTP	2	66.7	1	33.3	
Tumor size, median (range)		2.0	(0.3–12.0)	2.0	(1.2–4.0)	0.689^d^
Grade Type	Angioinvasive	1	100.0	0	0.0	–
	Classic	21	77.8	6	22.2	
	Follicular	5	71.4	2	28.6	
	High grade	1	100.0	0	0.0	
	Minimally invasive	1	100.0	0	0.0	
	NA	4	80.0	1	20.0	
	Well differentiated	1	100.0	0	0.0	
	Widely invasive	2	100.0	0	0.0	
T-stage	T1a	11	100.0	0	0.0	–
	T1b	5	71.4	2	28.6	
	T2	5	62.5	3	37.5	
	T3a	7	77.8	2	22.2	
	T3b	4	80.0	1	20.0	
N-stage^†^	N0	11	84.6	2	15.4	0.202^b^
	N1	7	58.3	5	41.7	
	Nx^†^	15	88.2	2	11.8	
Focality	Unifocal	27	81.8	6	18.2	0.613
	Multifocal	9	75.0	3	25.0	
Laterality	Unilateral	28	80.0	7	20.0	1.000^b^
	Isthmus	2	100.0	0	0.0	
	Bilateral	6	75.0	2	25.0	
Lymph Nodes	Negative	11	84.6	2	15.4	0.202^b^
	Positive	7	58.3	5	41.7	
Family History	No	10	71.4	4	28.6	0.461
	Yes	22	81.5	5	18.5	
Previous cancer	No	24	75.0	8	25.0	0.313
	Yes	9	90.0	1	10.0	
Chemotherapy	No	29	76.3	9	23.7	0.561^b^
	Yes	4	100.0	0	0.0	
Radiotherapy	No	28	75.7	9	24.3	0.567^b^
	Yes	5	100.0	0	0.0	
Obesity	Normal	10	100.0	0	0.0	0.029^b*^
	Overweight	10	90.9	1	9.1	
	Obese	13	61.9	8	38.1	
Chronic thyroiditis (e.g., Hashimoto's thyroiditis)	No	31	81.6	7	18.4	0.277^b^
	Yes	3	60.0	2	40.0	
Covid 19	No	25	80.6	6	19.4	0.582
	Yes	8	72.7	3	27.3	
Covid 19 vaccine	No	12	85.7	2	14.3	0.425
	Yes	21	75.0	7	25.0	

		HPV- 6& 11				*p*-value
		Negative (n = 30)		Positive (n = 15)		
		n	(%)	n	(%)	
Age, mean (SD)		43	11	48	12	0.138^c^
Gender	Male	8	72.7	3	27.3	0.624
	Female	22	64.7	12	35.3	
Tumor type	Papillary carcinoma	23	65.7	12	34.3	–
	Follicular carcinoma	4	100.0	0	0.0	
	Medullary carcinoma	1	100.0	0	0.0	
	Hurthle cell carcinoma	1	100.0	0	0.0	
	Anaplastic carcinoma	0	0.0	1	100.0	
	NIFTP	1	33.3	2	66.7	
Tumor size, median (range)		2.0	(0.3–6.5)	1.5	(0.3–12.0)	0.567^d^
Grade Type	Angioinvasive	1	100.0	0	0.0	–
	Classic	16	59.3	11	40.7	
	Follicular	6	85.7	1	14.3	
	High grade	0	0.0	1	100.0	
	Minimally invasive	1	100.0	0	0.0	
	NA	3	60.0	2	40.0	
	Well differentiated	1	100.0	0	0.0	
	Widely invasive	2	100.0	0	0.0	
T-stage	T1a	8	72.7	3	27.3	–
	T1b	4	57.1	3	42.9	
	T2	6	75.0	2	25.0	
	T3a	6	66.7	3	33.3	
	T3b	3	60.0	2	40.0	
N-stage^†^	N0	9	69.2	4	30.8	0.688^b^
	N1	7	58.3	5	41.7	
	Nx^†^	11	64.7	6	35.3	
Focality	Unifocal	24	72.7	9	27.3	0.153
	Multifocal	6	50.0	6	50.0	
Laterality	Unilateral	26	74.3	9	25.7	0.099^b^
	Isthmus	1	50.0	1	50.0	
	Bilateral	3	37.5	5	62.5	
Lymph Nodes	Negative	9	69.2	4	30.8	0.688^b^
	Positive	7	58.3	5	41.7	
Family History	No	10	71.4	4	28.6	0.588
	Yes	17	63.0	10	37.0	
Previous cancer	No	23	71.9	9	28.1	0.200
	Yes	5	50.0	5	50.0	
Chemotherapy	No	26	68.4	12	31.6	0.590^b^
	Yes	2	50.0	2	50.0	
Radiotherapy	No	26	70.3	11	29.7	0.313^b^
	Yes	2	40.0	3	60.0	
Obesity	Normal	9	90.0	1	10.0	0.043^b*^
	Overweight	9	81.8	2	18.2	
	Obese	10	47.6	11	52.4	
Chronic thyroiditis (e.g., Hashimoto's thyroiditis)	No	26	68.4	12	31.6	0.324^b^
	Yes	2	40.0	3	60.0	
Covid 19	No	22	71.0	9	29.0	0.321
	Yes	6	54.5	5	45.5	
Covid 19 vaccine	No	10	71.4	4	28.6	0.643
	Yes	18	64.3	10	35.7	

Raw percentages are shown

^†^ Nx was not included in comparison being of unknown state (not assessed)

^a^ Chi-square test

^b^ Fisher’s exact test

^C^ independent t-test

^d^ Mann–Whitney U test

**Table 4:** Association of polyoma virus and JCV with clinicopathological characteristics in thyroid cancer patients

		Polyoma virus				*p*-value
		Negative (n = 28)		Positive (n = 17)		
		n	(%)	n	(%)	
Age, mean (SD)		42	11	49	11	0.041^*c^
Gender	Male	6	54.5	5	45.5	0.546
	Female	22	64.7	12	35.3	
Tumor type	Papillary carcinoma	22	62.9	13	37.1	–
	Follicular carcinoma	2	50.0	2	50.0	
	Medullary carcinoma	1	100.0	0	0.0	
	Hurthle cell carcinoma	1	100.0	0	0.0	
	Anaplastic carcinoma	0	0.0	1	100.0	
	NIFTP	2	66.7	1	33.3	
Tumor size, median (range)		1.5	(0.3–6.0)	2.0	(0.6–12.0)	0.239^d^
Grade Type	Angioinvasive	1	100.0	0	0.0	–
	Classic	17	63.0	10	37.0	
	Follicular	5	71.4	2	28.6	
	High grade	0	0.0	1	100.0	
	Minimally invasive	0	0.0	1	100.0	
	NA	4	80.0	1	20.0	
	Well differentiated	0	0.0	1	100.0	
	Widely invasive	1	50.0	1	50.0	
T-stage	T1a	8	72.7	3	27.3	-
	T1b	5	71.4	2	28.6	
	T2	5	62.5	3	37.5	
	T3a	5	55.6	4	44.4	
	T3b	2	40.0	3	60.0	
N-stage^†^	N0	6	46.2	7	53.8	0.695^b^
	N1	7	58.3	5	41.7	
	Nx^†^	13	76.5	4	23.5	
Focality	Unifocal	21	63.6	12	36.4	0.746
	Multifocal	7	58.3	5	41.7	
Laterality	Unilateral	22	62.9	13	37.1	–
	Isthmus	1	50.0	1	50.0	
	Bilateral	5	62.5	3	37.5	
Lymph Nodes	Negative	6	46.2	7	53.8	0.543
	Positive	7	58.3	5	41.7	
Family History	No	8	57.1	6	42.9	0.548
	Yes	18	66.7	9	33.3	
Previous cancer	No	21	65.6	11	34.4	0.374
	Yes	5	50.0	5	50.0	
Chemotherapy	No	25	65.8	13	34.2	0.146^b^
	Yes	1	25.0	3	75.0	
Radiotherapy	No	24	64.9	13	35.1	0.352^b^
	Yes	2	40.0	3	60.0	
Obesity	Normal	6	60.0	4	40.0	1.000^b^
	Overweight	7	63.6	4	36.4	
	Obese	13	61.9	8	38.1	
Chronic thyroiditis (e.g., Hashimoto's thyroiditis)	No	25	65.8	13	34.2	0.344^b^
	Yes	2	40.0	3	60.0	
Covid 19	No	21	67.7	10	32.3	0.191
	Yes	5	45.5	6	54.5	
Covid 19 vaccine	No	10	71.4	4	28.6	0.369
	Yes	16	57.1	12	42.9	

	JCV					*p*-value
		Negative (n = 32)		Positive (n = 13)		
		n	(%)	n	(%)	
Age, mean (SD)		42	11	51	12	0.011^*c^
Gender	Male	6	54.5	5	45.5	0.163
	Female	26	76.5	8	23.5	
Tumor type	Papillary carcinoma	26	74.3	9	25.7	–
	Follicular carcinoma	2	50.0	2	50.0	
	Medullary carcinoma	1	100.0	0	0.0	
	Hurthle cell carcinoma	1	100.0	0	0.0	
	Anaplastic carcinoma	0	0.0	1	100.0	
	NIFTP	2	66.7	1	33.3	
Tumor size, median (range)		1.5	(0.3–6.0)	2.0	(1.0–12.0)	0.169^d^
Grade Type	Angioinvasive	1	100.0	0	0.0	–
	Classic	20	74.1	7	25.9	
	Follicular	6	85.7	1	14.3	
	High grade	0	0.0	1	100.0	
	Minimally invasive	0	0.0	1	100.0	
	NA	4	80.0	1	20.0	
	Well differentiated	0	0.0	1	100.0	
	Widely invasive	1	50.0	1	50.0	
T-stage	T1a	9	81.8	2	18.2	–
	T1b	6	85.7	1	14.3	
	T2	6	75.0	2	25.0	
	T3a	5	55.6	4	44.4	
	T3b	3	60.0	2	40.0	
N-stage^†^	N0	6	46.2	7	53.8	0.141^b^
	N1	9	75.0	3	25.0	
	Nx^†^	15	88.2	2	11.8	
Focality	Unifocal	23	69.7	10	30.3	0.729
	Multifocal	9	75.0	3	25.0	
Laterality	Unilateral	24	68.6	11	31.4	1.000^b^
	Isthmus	2	100.0	0	0.0	
	Bilateral	6	75.0	2	25.0	
Lymph Nodes	Negative	6	46.2	7	53.8	0.141
	Positive	9	75.0	3	25.0	
Family History	No	9	64.3	5	35.7	0.355
	Yes	21	77.8	6	22.2	
Previous cancer	No	24	75.0	8	25.0	0.359
	Yes	6	60.0	4	40.0	
Chemotherapy	No	29	76.3	9	23.7	0.063^b^
	Yes	1	25.0	3	75.0	
Radiotherapy	No	28	75.7	9	24.3	0.131^b^
	Yes	2	40.0	3	60.0	
Obesity	Normal	6	60.0	4	40.0	0.610^b^
	Overweight	8	72.7	3	27.3	
	Obese	16	76.2	5	23.8	
Chronic thyroiditis (e.g., Hashimoto's thyroiditis)	No	28	73.7	10	26.3	0.608^b^
	Yes	3	60.0	2	40.0	
Covid 19	No	22	71.0	9	29.0	0.912
	Yes	8	72.7	3	27.3	
Covid 19 vaccine	No	10	71.4	4	28.6	1.000
	Yes	20	71.4	8	28.6	

Raw percentages are shown

^†^ Nx was not included in comparison being of unknown state (not assessed)

^a^ Chi-square test

^b^ Fisher’s exact test

^C^ independent t-test

^d^ Mann–Whitney U test

**Table 5:** Association of EBV and BKV with clinicopathological characteristics in TC patients

	EBV					*p*-value^a^
		Negative (n = 25)			Positive (n = 20)	
		n	(%)	n	(%)	
Age, mean (SD)		43	11	46	12	0.369^c^
Gender	Male	5	45.5	6	54.5	0.438
	Female	20	58.8	14	41.2	
Tumor type	Papillary carcinoma	19	54.3	16	45.7	–
	Follicular carcinoma	3	75.0	1	25.0	
	Medullary carcinoma	0	0.0	1	100.0	
	Hurthle cell carcinoma	1	100.0	0	0.0	
	Anaplastic carcinoma	0	0.0	1	100.0	
	NIFTP	2	66.7	1	33.3	
Tumor size, median (range)		2.0	(0.3–6.5)	1.8	(0.3–12.0)	0.776^d^
Grade Type	Angioinvasive	1	100.0	0	0.0	–
	Classic	16	59.3	11	40.7	
	Follicular	3	42.9	4	57.1	
	High grade	0	0.0	1	100.0	
	Minimally invasive	1	100.0	0	0.0	
	Well differentiated	0	0.0	1	100.0	
	Widely invasive	1	50.0	1	50.0	
T-stage	T1a	7	63.6	4	36.4	–
	T1b	2	28.6	5	71.4	
	T2	5	62.5	3	37.5	
	T3a	6	66.7	3	33.3	
	T3b	2	40.0	3	60.0	
N-stage^†^	N0	6	46.2	7	53.8	0.411^b^
	N1	3	25.0	9	75.0	
	Nx^†^	15	88.2	2	11.8	
Focality	Unifocal	19	57.6	14	42.4	0.651
	Multifocal	6	50.0	6	50.0	
Laterality	Unilateral	18	51.4	17	48.6	-
	Isthmus	2	100.0	0	0.0	
	Bilateral	5	62.5	3	37.5	
Lymph Nodes	Negative	6	46.2	7	53.8	0.411^b^
	Positive	3	25.0	9	75.0	
Family History	No	4	28.6	10	71.4	0.020*
	Yes	18	66.7	9	33.3	
Previous cancer	No	18	56.3	14	43.8	0.729
	Yes	5	50.0	5	50.0	
Chemotherapy	No	21	55.3	17	44.7	1.000^b^
	Yes	2	50.0	2	50.0	
Radiotherapy	No	20	54.1	17	45.9	1.000^b^
	Yes	3	60.0	2	40.0	
Obesity	Normal	6	60.0	4	40.0	0.717^b^
	Overweight	7	63.6	4	36.4	
	Obese	10	47.6	11	52.4	
Chronic thyroiditis (e.g., Hashimoto's thyroiditis)	No	22	57.9	16	42.1	0.640^b^
	Yes	2	40.0	3	60.0	
Covid 19	No	17	54.8	14	45.2	0.987
	Yes	6	54.5	5	45.5	
Covid 19 vaccine	No	8	57.1	6	42.9	0.826
	Yes	15	53.6	13	46.4	

	BKV					*p*-value
		Negative (n = 37)		Positive (n = 8)		
		n	(%)	n	(%)	
Age, mean (SD)		43	11	52	13	0.057^c^
Gender	Male	9	81.8	2	18.2	0.968
	Female	28	82.4	6	17.6	
Tumor type	Papillary carcinoma	30	85.7	5	14.3	–
	Follicular carcinoma	3	75.0	1	25.0	
	Medullary carcinoma	1	100.0	0	0.0	
	Hurthle cell carcinoma	1	100.0	0	0.0	
	Anaplastic carcinoma	0	0.0	1	100.0	
	NIFTP	2	66.7	1	33.3	
Tumor size, median (range)		1.5	(0.3–6.5)	3.3	(1.5–12.0)	0.030^*d^
Grade Type	Angioinvasive	1	100.0	0	0.0	–
	Classic	23	85.2	4	14.8	
	Follicular	7	100.0	0	0.0	
	High grade	0	0.0	1	100.0	
	Minimally invasive	0	0.0	1	100.0	
	Well differentiated	0	0.0	1	100.0	
	Widely invasive	2	100.0	0	0.0	
T-stage	T1a	11	100.0	0	0.0	–
	T1b	7	100.0	0	0.0	
	T2	6	75.0	2	25.0	
	T3a	7	77.8	2	22.2	
	T3b	3	60.0	2	40.0	
N-stage^†^	N0	10	76.9	3	23.1	1.000^b^
	N1	9	75.0	3	25.0	
	Nx^†^	16	94.1	1	5.9	
Focality	Unifocal	26	78.8	7	21.2	0.318
	Multifocal	11	91.7	1	8.3	
Laterality	Unilateral	28	80.0	7	20.0	1.000^b^
	Isthmus	2	100.0	0	0.0	
	Bilateral	7	87.5	1	12.5	
Lymph Nodes	Negative	10	76.9	3	23.1	1.000^b^
	Positive	9	75.0	3	25.0	
Family History	No	12	85.7	2	14.3	1.000^b^
	Yes	22	81.5	5	18.5	
Previous cancer	No	28	87.5	4	12.5	0.195
	Yes	7	70.0	3	30.0	
Chemotherapy	No	33	86.8	5	13.2	0.123^b^
	Yes	2	50.0	2	50.0	
Radiotherapy	No	32	86.5	5	13.5	0.188^b^
	Yes	3	60.0	2	40.0	
Obesity	Normal	8	80.0	2	20.0	0.756^b^
	Overweight	10	90.9	1	9.1	
	Obese	17	81.0	4	19.0	
Chronic thyroiditis (e.g., Hashimoto's thyroiditis)	No	32	84.2	6	15.8	1.000^b^
	Yes	4	80.0	1	20.0	
Covid 19	No	26	83.9	5	16.1	0.875
	Yes	9	81.8	2	18.2	
Covid 19 vaccine	No	13	92.9	1	7.1	0.242^b^
	Yes	22	78.6	6	21.4	

Raw percentages are shown

^†^ Nx was not included in comparison being of unknown state

^a^ Chi-square test

^b^ Fisher’s exact test

^C^ independent t-test

^d^ Mann–Whitney U test

### Prevalence of HPV, EBV, and polyomaviruses across malignant, benign, and normal groups

As shown in Table 6, HPV common, EBV, and polyomaviruses, particularly JC virus, were detected at significantly lower frequencies in the normal group when compared to malignant and benign groups (*p* = 0.030, 0.001, and 0.019, respectively). HPV-18 and HPV-16, high risk subtypes, were more prevalent in malignant cases compared to benign one, though did not reach significance. Moreover, when comparing viral prevalence between malignant and benign groups, negativity of polyomaviruses was significantly lower in the benign group (*p*-value = 0.015) as shown in Table 7.

**Table 6:** Prevalence of HPV, EBV, and polyomaviruses across malignant, benign, and normal groups

		Group						*p*-value
		Malignant (n = 45)		Benign (n = 25)		Normal (n = 10)		
		n	(%)	n	(%)	n	(%)	
HPV common	Negative	25	55.6	16	64.0	10	100.0	0.030^**^
	Positive	20	44.4	9	36.0	0	0.0	
HPV-18	Negative	33	73.3	18	72.0	10	100.0	0.167
	Positive	12	26.7	7	28.0	0	0.0	
HPV-16	Negative	36	80.0	18	72.0	10	100.0	0.174
	Positive	9	20.0	7	28.0	0	0.0	
HPV-611	Negative	30	66.7	19	76.0	10	100.0	0.091
	Positive	15	33.3	6	24.0	0	0.0	
HPV-58	Negative	45	100.0	25	100.0	10	100.0	–
	Positive	0	0.0	0	0.0	0	0.0	
HPV-33	Negative	45	100.0	25	100.0	10	100.0	–
	Positive	0	0.0	0	0.0	0	0.0	
EBV	Negative	25	55.6	15	60.0	10	100.0	0.030^**^
	Positive	20	44.4	10	40.0	0	0.0	
Polyoma Viruses	Negative	28	62.2	8	32.0	10	100.0	0.001^**^
	Positive	17	37.8	17	68.0	0	0.0	
JC	Negative	32	71.1	13	52.0	10	100.0	0.019^**^
	Positive	13	28.9	12	48.0	0	0.0	
BK	Negative	37	82.2	19	76.0	10	100.0	0.240
	Positive	8	17.8	6	24.0	0	0.0	

^*^Statistically significant at < 0.05 level

^a^Chi-square test

^**^Normal group is significantly different from both malignant and benign groups

**Table 7:** Prevalence of HPV, EBV, and polyomaviruses between malignant and benign groups

	Group					
		Malignant (n = 45)		Benign (n = 25)		*p*-value ^a^
		n	(%)	n	(%)	
HPV common	Negative	25	55.6	16	64.0	0.492
	Positive	20	44.4	9	36.0	
HPV-18	Negative	33	73.3	18	72.0	0.904
	Positive	12	26.7	7	28.0	
HPV-16	Negative	36	80.0	18	72.0	0.445
	Positive	9	20.0	7	28.0	
HPV-611	Negative	30	66.7	19	76.0	0.414
	Positive	15	33.3	6	24.0	
HPV-58	Negative	45	100.0	25	100.0	–
	Positive	0	0.0	0	0.0	
HPV-33	Negative	45	100.0	25	100.0	–
	Positive	0	0.0	0	0.0	
EBV	Negative	25	55.6	15	60.0	0.719
	Positive	20	44.4	10	40.0	
Polyoma Viruses	Negative	28	62.2	8	32.0	0.015^*^
	Positive	17	37.8	17	68.0	
JC	Negative	32	71.1	13	52.0	0.110
	Positive	13	28.9	12	48.0	
BK	Negative	37	82.2	19	76.0	0.533
	Positive	8	17.8	6	24.0	

^a^Chi-square test

### Association of polyomavirus and BKV with clinical characteristics in patients with benign thyroid tumors

**Fig. 3:**
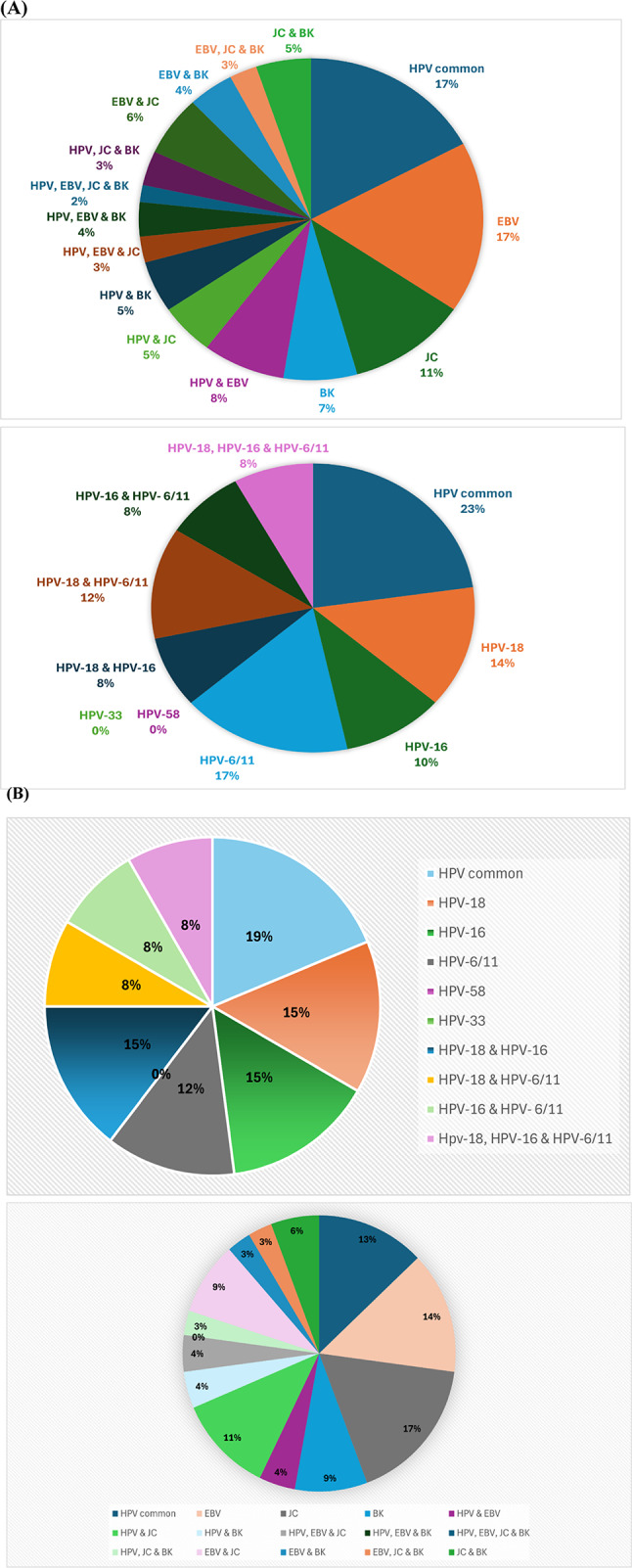
Distribution of Single and Multiple Viral Presence (HPV, EBV, JCV, and BKV) in **A** Malignant and **B** Benign Thyroid Tumors

As shown in Table 8, a statistically significant association was detected with Hashimoto thyroiditis, all patients with Hashimoto’s were negative for polyomavirus (*p*-value = 0.020). Additionally, age was significantly higher in BK-positive patients, and COVID-19 vaccination was significantly associated with absence of BKV (*p*-value = 0.030 and 0.046, respectively). Of interest, multivariate logistic regression was performed to assess the association between BKV and the significantly detected variables in univariate analysis level (age and COVID-19 vaccination status). However, none of them remained significantly associated with BKV in the multivariate model. Moreover, when comparing the clinicopathological features of TC patients based on viral co-presence status, whether it is single viral presence, multiple viral presence, or undetectable viral presence, no statistically significant associations were identified (supplementary Table 1). Figure 3 represents the distribution of single and multiple viral presence (HPV, EBV, JCV, and BKV) in malignant and benign thyroid tumors.

**Table 8:** Association of Polyomavirus and BKV with Clinical Characteristics in Patients with Benign Thyroid Tumors

		Polyoma virus				*p*-value^b^
		Negative (n = 8)		Positive (n = 17)		
		n	(%)	n	(%)	
Age, mean (SD)		46	8	45	14	0.825
Gender	Male	0	0.0	1	100.0	1.000
	Female	8	33.3	16	66.7	
Family History	No	3	30.0	7	70.0	1.000
	Yes	4	30.8	9	69.2	
Previous cancer	No	4	23.5	13	76.5	0.318
	Yes	3	50.0	3	50.0	
Chemotherapy	No	6	28.6	15	71.4	0.526
	Yes	1	50.0	1	50.0	
Radiotherapy	No	6	28.6	15	71.4	0.526
	Yes	1	50.0	1	50.0	
Obesity	Normal	0	0.0	2	100.0	–
	Overweight	4	36.4	7	63.6	
	Obese	2	22.2	7	77.8	
Hashimoto thyroiditis	No	4	20.0	16	80.0	0.020*
	Yes	3	100.0	0	0.0	
Covid 19	No	5	27.8	13	72.2	–
	Yes	0	0.0	3	100.0	
Covid 19 vaccine	No	4	33.3	8	66.7	0.338
	Yes	1	11.1	8	88.9	

		BK				*p*-value^b^
		Negative (n = 19)		Positive (n = 6)		
		n	(%)	n	(%)	
Age, mean (SD)		42	10	54	14	0.030
Gender	Male	0	0.0	1	100.0	0.240
	Female	19	79.2	5	20.8	
Family History	No	7	70.0	3	30.0	1.000
	Yes	10	76.9	3	23.1	
Previous cancer	No	11	64.7	6	35.3	–
	Yes	6	100.0	0	0.0	
Chemotherapy	No	15	71.4	6	28.6	1.000
	Yes	2	100.0	0	0.0	
Radiotherapy	No	15	71.4	6	28.6	1.000
	Yes	2	100.0	0	0.0	
Obesity	Normal	2	100.0	0	0.0	–
	Overweight	8	72.7	3	27.3	
	Obese	6	66.7	3	33.3	
Hashimoto thyroiditis	No	14	70.0	6	30.0	0.539
	Yes	3	100.0	0	0.0	
Covid 19	No	13	72.2	5	27.8	1.000
	Yes	2	66.7	1	33.3	
Covid 19 vaccine	No	11	91.7	1	8.3	0.046*
	Yes	4	44.4	5	55.6	

Raw percentages are shown

^b^ Fisher’s exact test

^C^ Independent t-test

## Discussion

The present study investigated the prevalence of human papillomavirus (HPV), Epstein–Barr virus (EBV), and polyomaviruses in thyroid tumors. It also examined their associations with clinicopathological characteristics. Our findings detected significantly reduced frequencies of HPV, EBV, and polyomaviruses, particularly JCV, in normal group when compared to malignant and benign groups. Of note, significant associations were found between the presence of these viruses and some clinicopathological characteristics, suggesting that viral presence might affect tumor behavior.

It is well established that the interplay between obesity-induced metabolic stress and chronic inflammation creates a microenvironment suitable for tumor initiation and progression (Ramos-Nino 2013). In this context, we propose that viral infections may initiate oncogenic processes in metabolically stressed environments, based on our observed association between HPV subtypes (HPV-16, HPV-6, and HPV-11) and obesity.

Similarly, the association of BKV with larger tumor size observed in our study agrees with existing evidence that certain viral infections may disrupt the cellular regulatory mechanisms and promote tumor growth (Damian 2025). Thus, this finding might indicate a more aggressive role of this virus, suggesting that viral profiling could guide surgical planning decision. In addition, our finding that polyomaviruses, especially JCV, are associated with older age, and that EBV is associated with family history of cancer, underlines the complex role of viruses in influencing tumor behavior and cancer risk.

Our findings come in agreement with an accumulating body of evidence suggesting that viral presence may contribute to the development of thyroid cancers. Several studies have also reported the presence of HPV DNA in TC tissues, especially in PTCs, which points to a direct role of HPV in thyroid carcinogenesis (Meng 2025). In a study by Yang et al., data from over 3000 TC patients and 9186 matched controls revealed a significant difference in the prevalence of prior HPV infection between TC patients (15.3%) and controls (7.6%). In their study, patients infected with HPV were more 2.199 times likely to develop TC than those without the infection (Yang et al. 2024). Also, our findings agreed with Dialameh et al. who reported the presence of HPV in 13.4% of PTC samples and 3.8% of benign thyroid nodules, with no HPV detected in the adjacent normal tissues (Archin Dialameh et al. 2021).

The role of EBV in thyroid carcinogenesis is still controversial, though several studies reported its presence in thyroid tumors. Moghoofei et al. suggested that EBV might have a potential oncogenic role in thyroid carcinogenesis, based on higher expression levels of specific EBV genes. In the former study, EBV DNA was detected in 71.9% of TC samples (Moghoofei et al. 2019). Likewise, another study identified EBV sequences, including EBNA2 and LMP1, in both thyroid nodules and adjacent normal tissues. EBNA2 was detected in 90% of both TC samples and adjacent normal thyroid tissue samples, whereas LMP1 was found in 50% of TC and 46.7% of the adjacent tissues (Stamatiou et al. 2015). Additionally, another study detected EBNA1 in 65.8% of PTC samples, especially among younger ages (Homayouni et al. 2017). Also, Mostafaei et al. reported that EBV positivity was significantly higher in TC patients compared to healthy controls (Mostafaei et al. 2020b). Furthermore, viral contribution to TC initiation and progression was supported in a meta-analysis by Mostafaei et al., particularly involving EBV and BKV (Mostafaei et al. 2020a). In contrast, no evidence of EBV involvement was found in a study on Warthin-like PTC, revealing that viral contribution may vary depending on histological subtypes (Ludvíková et al. 2001).

While some studies have reported EBV in the majority of PTC (Stamatiou et al. 2015) and in all papillary and undifferentiated carcinomas (Shimakage et al. 2003), others such as a study in southern Japan, demonstrated the viral absence in all the TC cases examined by EBER-1 in situ hybridization (Kijima et al. 2001). These contradictory findings reveal that, although EBV may contribute to thyroid carcinogenesis, its involvement is likely depends on host–virus interactions and additional co-factors (Stamatiou et al. 2016).

The previous studies collectively suggest a potential role of JCV in several cancers, including lung, central nervous system, breast, medulloblastoma, and colorectal cancers, through viral DNA detection and viral oncoproteins expression in tumor tissues (Del Valle et al. 2002; Enam et al. 2002; Sadeghi et al. 2015; Sinagra et al. 2017; Zheng et al. 2021). Few studies have investigated the link between polyomaviruses and TC. It was reported in three of those studies that DNA sequences of simian vacuolating virus 40 (SV40) were detected in thyroid tumors (Ghanghareh et al. 2021). Additionally, Karimi et al. examined JCV in a sizable cohort of 1057 patients with PTC and their adjacent non-cancerous tissues and detected JCV DNA in 10.8% of all samples (Karimi et al. 2022). In the present study, JCV negativity in the normal group was significantly higher compared to the benign (52%) and malignant (71.1%) groups. polyomaviruses negativity was also significantly higher in the malignant group (62.6%) than in the benign (32%) group. Although not statistically significant, positivity of JCV and BKV was detected in malignant and benign thyroid tissues. Also, other researchers using post-operative thyroid samples reported the presence of BKV in malignant lesions and multinodular hyperplasia lesions, with BKV DNA found in nearly 60% of nodular tissues and 43.3% of adjacent normal tissues among a group of 30 patients with cancer and multinodular hyperplasia. In PTC cases, BKV was detected in 57.1% of tumors and 42.8% of adjacent tissues (Stamatiou et al. 2015).

In summary, our results support the increasing evidence that HPV, EBV, and polyomaviruses may play a role in thyroid carcinogenesis, each exhibiting unique association with certain clinicopathological characteristics, which in turn may influence tumor behavior and patient risk for cancer development.

## Conclusion

The current study detected HPV, EBV, and polyomaviruses prevalence in both malignant and benign thyroid tissues, with significant associations to some clinicopathological features. The observed association between viral presence, obesity, family cancer history, and tumor size shed the light on the potential role of these viruses in thyroid carcinogenesis. Our results cannot confirm causation, however, in line with earlier studies, point to the need for larger studies to clarify the possible role of viral infections in TC development.

### Study limitations and future directions

This study has some limitations, including a relatively small sample size (n = 45 malignant cases), particularly for rare histological subtypes the study doesn't consider all potential variables, such as environmental exposures or individual immune profiles, and possible variability in viral detection methods. Larger studies, including different populations and histological subtypes are needed to confirm these findings. Longitudinal studies could help determine whether viral infections influence TC progression and explore the molecular mechanisms behind these associations. Furthermore, investigating the presence of multiple viruses and the possible effects of co-infections that could reveal the probable synergistic influences on tumor behavior.

## Supplementary Information

Below is the link to the electronic supplementary material.

Supplementary Material 1

## Data Availability

No datasets were generated or analysed during the current study.
