# Supplementary material for: Molecular detection of HPV, EBV, and polyomaviruses in thyroid tumors and their clinicopathological relevance
Source: J Cancer Res Clin Oncol. 2025 Oct 20;151(12):298. doi: 10.1007/s00432-025-06328-1 (PMC12537628; doi:10.1007/s00432-025-06328-1)
Supplement: Supplementary file 1 — Supplementary Material 1 [file 432_2025_6328_MOESM1_ESM.docx]

**Supplementary Table 1: Relation between Viral Co-presence Status and Clinicopathological Characteristics of TC Patients**

|  | | Viral Copresence | | | | | |  |
| --- | --- | --- | --- | --- | --- | --- | --- | --- |
|  |  | Single  (n=13) | | Multiple  (n=21) | | Negative all  (n=11) | |  |
|  |  | n | % | n | % | n | % | p-value ^a^ |
| Age, mean (SD) |  | 42 | 13 | 48 | 11 | 40 | 10 | 0.136 ^b^ |
| Gender | Male | 4 | 36.4% | 6 | 54.5% | 1 | 9.1% | 0.448 |
|  | Female | 9 | 26.5% | 15 | 44.1% | 10 | 29.4% |  |
| Tumor type | Papillary carcinoma | 7 | 20.0% | 18 | 51.4% | 10 | 28.6% | - |
|  | Follicular carcinoma | 3 | 75.0% | 1 | 25.0% | 0 | 0.0% |  |
|  | Medullary carcinoma | 1 | 100.0% | 0 | 0.0% | 0 | 0.0% |  |
|  | Hurthle cell carcinoma | 0 | 0.0% | 0 | 0.0% | 1 | 100.0% |  |
|  | Anaplastic carcinoma | 0 | 0.0% | 1 | 100.0% | 0 | 0.0% |  |
|  | NIFTP | 2 | 66.7% | 1 | 33.3% | 0 | 0.0% |  |
| Grade Type | Angioinvasive | 1 | 100.0% | 0 | 0.0% | 0 | 0.0% | - |
|  | Classic | 6 | 22.2% | 14 | 51.9% | 7 | 25.9% |  |
|  | Follicular | 1 | 14.3% | 3 | 42.9% | 3 | 42.9% |  |
|  | High grade | 0 | 0.0% | 1 | 100.0% | 0 | 0.0% |  |
|  | Minimally invasive | 0 | 0.0% | 1 | 100.0% | 0 | 0.0% |  |
|  | NA | 3 | 60.0% | 1 | 20.0% | 1 | 20.0% |  |
|  | Well differentiated | 0 | 0.0% | 1 | 100.0% | 0 | 0.0% |  |
|  | Widely invasive | 2 | 100.0% | 0 | 0.0% | 0 | 0.0% |  |
| Tumor size, median (range) |  | 1.5 | (0.3-6.5) | 2.0 | (0.6-12.0 | 2.5 | (0.3-6.0) | 0.658 ^c^ |
| T | T1a | 4 | 36.4% | 3 | 27.3% | 4 | 36.4% | - |
|  | T1b | 2 | 28.6% | 4 | 57.1% | 1 | 14.3% |  |
|  | T2 | 2 | 25.0% | 5 | 62.5% | 1 | 12.5% |  |
|  | T3a | 1 | 11.1% | 5 | 55.6% | 3 | 33.3% |  |
|  | T3b | 2 | 40.0% | 2 | 40.0% | 1 | 20.0% |  |
| N | N0 | 4 | 30.8% | 7 | 53.8% | 2 | 15.4% | - |
|  | N1 | 4 | 33.3% | 8 | 66.7% | 0 | 0.0% |  |
|  | Nx | 4 | 23.5% | 5 | 29.4% | 8 | 47.1% |  |
| Focality | Unifocal | 12 | 36.4% | 13 | 39.4% | 8 | 24.2% | 0.143 |
|  | Multifocal | 1 | 8.3% | 8 | 66.7% | 3 | 25.0% |  |
| Laterality | Unilateral | 13 | 37.1% | 14 | 40.0% | 8 | 22.9% | - |
|  | Isthmus | 0 | 0.0% | 1 | 50.0% | 1 | 50.0% |  |
|  | Bilateral | 0 | 0.0% | 6 | 75.0% | 2 | 25.0% |  |
| Lymph Nodes | Negative | 4 | 30.8% | 7 | 53.8% | 2 | 15.4% | - |
|  | Positive | 4 | 33.3% | 8 | 66.7% | 0 | 0.0% |  |
| Family History | No | 4 | 28.6% | 8 | 57.1% | 2 | 14.3% | 0.484 |
|  | Yes | 7 | 25.9% | 11 | 40.7% | 9 | 33.3% |  |
| Previous cancer | No | 9 | 28.1% | 13 | 40.6% | 10 | 31.3% | 0.425 |
|  | Yes | 3 | 30.0% | 6 | 60.0% | 1 | 10.0% |  |
| Obesity | Normal | 4 | 40.0% | 3 | 30.0% | 3 | 30.0% | - |
|  | Overweight | 4 | 36.4% | 3 | 27.3% | 4 | 36.4% |  |
|  | Obese | 4 | 19.0% | 13 | 61.9% | 4 | 19.0% |  |
| Chemotherapy | No | 11 | 28.9% | 16 | 42.1% | 11 | 28.9% | 0.563 |
|  | Yes | 1 | 25.0% | 3 | 75.0% | 0 | 0.0% |  |
| Radiotherapy | No | 11 | 29.7% | 15 | 40.5% | 11 | 29.7% | 0.352 |
|  | Yes | 1 | 20.0% | 4 | 80.0% | 0 | 0.0% |  |
| Chronic thyroiditis (e.g., Hashimoto's thyroiditis) | No | 11 | 28.9% | 17 | 44.7% | 10 | 26.3% | 1.000 |
|  | Yes | 1 | 20.0% | 3 | 60.0% | 1 | 20.0% |  |
| covid 19 | No | 9 | 29.0% | 14 | 45.2% | 8 | 25.8% | 1.000 |
|  | Yes | 3 | 27.3% | 5 | 45.5% | 3 | 27.3% |  |
| covid 19 vaccine | No | 5 | 35.7% | 5 | 35.7% | 4 | 28.6% | 0.702 |
|  | Yes | 7 | 25.0% | 14 | 50.0% | 7 | 25.0% |  |
